# A Lightweight Cipher Based on Salsa20 for Resource-Constrained IoT Devices

**DOI:** 10.3390/s18103326

**Published:** 2018-10-04

**Authors:** Evangelina Lara, Leocundo Aguilar, Jesús A. García, Mauricio A. Sanchez

**Affiliations:** Facultad de Ciencias Químicas e Ingeniería, Universidad Autónoma de Baja California, Tijuana, BC 22390, Mexico; evangelina.lara@uabc.edu.mx (E.L.); garcia.jesus@uabc.edu.mx (J.A.G.); mauricio.sanchez@uabc.edu.mx (M.A.S.)

**Keywords:** Internet of Things, cipher, Salsa20

## Abstract

The Internet of Things (IoT) paradigm envisions a world where everyday things interchange information between each other in a way that allows users to make smarter decisions in a given context. Even though IoT has many advantages, its characteristics make it very vulnerable to security attacks. Ciphers are a security primitive that can prevent some of the attacks; however, the constrained computing and energy resources of IoT devices impede them from implementing current ciphers. This article presents the stream cipher Generador de Bits Pseudo Aleatorios (GBPA) based on Salsa20 cipher, which is part of the eSTREAM project, but designed for resource-constrained IoT devices of Class 0. GBPA has lower program and data memory requirements compared with Salsa20 and lightweight ciphers. These properties allow low-cost resource-constrained IoT devices, 29.5% of the embedded systems in the market, to be able to implement a security service that they are currently incapable of, to preserve the user’s data privacy and protect the system from attacks that could damage it. For the evaluation of its output, three statistical test suites were used: NIST Statistical Test Suite (STS), DIEHARD and EACirc, with good results. The GBPA cipher provides security without having a negative impact on the computing resources of IoT devices.

## 1. Introduction

Society is moving towards a more connected world. The Internet of Things (IoT) is a technology whose goal is that everyday objects interact and exchange information with each other to accomplish a particular objective. With this simple idea, a wide range of applications is conceivable, which include: smart cities, smart houses, smart farming, industrial automation, security, medical services, entertainment, etc. IoT devices are physically composed of sensors, actuators, microcontrollers and transceivers, to accomplish their mission; and are capable of communicating and identifying each other through the Internet. Ideally, they are everywhere and always on; therefore, when notifying about the data perceived by their sensors and the state of their actuators, better knowledge of the current context can be achieved [1,2]. Cloud services are used by them to provide contextual data to users and to retrieve petitions to modify them; connectivity to the Internet makes them accessible from everywhere, and all the time; however, this empowerment also makes them very vulnerable because from anywhere in the world, they can be reached and attacked. If successful, an attacker could access private information such as personal, medical, financial or location data, and can also use actuators to bring severe damage to the system and even the user welfare [3]. An analysis of vulnerabilities and possible attacks is presented in [4,5,6]. Another concern is that, towards being ubiquitous, they are implemented as low-power devices with constrained resources, which creates a challenge in the implementation of security services. Due to constrained computing and energy resources, traditional security systems are not viable on them [7,8].

There are some proposals in the literature of ciphers for IoT, such as the block cipher ANU [9] and its modification, ANU-II [10]. In their articles, comparisons were presented of the computing requirements of the ANU ciphers against the ones of some well-known ciphers, such as PRESENT, KLEIN, PICCOLO, TWINE, KATAN, SIMON, AES, CLEFIA, LED, RECTANGLE, ZORRO and LiCi; showing that ANU ciphers have fewer requirements. However, the proposed algorithm in this article has even fewer: it requires less program memory (32.04% less than ANU and 15.01% less than ANU-II) and less data memory (98.47% less than ANU and 98.43% less than ANU-II).

There are also some proposed schemes based on public key encryption, such as [11,12]; however, the long key size and required computation of this type of cryptography makes it difficult to use on resource-constrained devices, because of their limited memory, power supply and network bandwidth.

A security primitive to prevent some of the attacks on IoT devices is encryption, which allows parties to communicate with confidentiality. An encryption algorithm, or cipher, takes a message, performs some transformations on it using a secret key and returns a ciphertext. The ciphertext can be sent through an insecure channel without worrying about unauthorized access to its content, because if an adversary captures the message, without the secret key, he/she would not be able to decrypt it to obtain the original data. This article presents the GBPA cipher, an acronym in Spanish for “Generador de Bits Pseudo Aleatorios” (“Pseudo-Random Bits Generator”). It is designed to provide confidentiality for the communication of resource-constrained IoT devices of Class 0 [13], corresponding to 29.5% of the embedded systems in the market (study conducted in 2017) [14], incapable of using current cipher algorithms. The proposal is based on the Salsa20 cipher, which is part of the portfolio of the eSTREAM project [15,16]. The purpose of this project was the identification of secure stream ciphers, and to be accepted in it, Salsa20 was under evaluation for four years by the international community. The GBPA cipher uses the Salsa20 core function, but to have a good performance on devices with constrained resources, it has fewer parameters, has a smaller memory footprint and requires fewer execution cycles. When comparing it with lightweight ciphers, lower program and data memory usage are shown. This allows low-cost resource-constrained IoT devices to be able to implement a security service to protect the user’s data privacy, the user’s welfare and the system from attacks that could damage or disrupt its functionality. GBPA has an output size of 128 bits and a key size of 96 bits and consists of 10 rounds of a lightweight add-rotate-xor function. Considering that the information sent by IoT devices is usually contextual data in the form of short length packets, the small output size of the GBPA cipher is appropriate for them because no unnecessary processor and memory usage is needed. The properties of GBPA makes it suitable to the restrictions of IoT devices, such as limited memory, processing and energy resources and low bandwidth. Analysis of the computing requirements of the algorithm along with the evaluation of the security of its output is presented in this document.

The rest of this document is organized as follows: Section 2 contains the background. In Section 3, the GBPA cipher is described. The evaluation of the security of the output of the algorithm and analysis of its computing requirements are in Section 4. A discussion is presented in Section 5. Finally, conclusions are given in Section 6.

## 2. Background

### 2.1. Symmetric Cipher Algorithms

A symmetric cipher consists of a set of encryption transformations $\left\{E_{k}:k\in K\right\}$ and a set of decryption transformations $\left\{D_{k}:k\in K\right\}$, where *k* is the encryption and decryption key. The encryption function takes as input a message and a key and returns as output a ciphertext, which is the message after doing some transformations on it with the key. To recover the original message, the ciphertext and the key are input to the decryption function, which usually consists of the encryption transformations performed backward. The original message cannot be recovered from a ciphertext without knowing the key; therefore, the key must be kept secret from unauthorized parties [17].

Symmetric ciphers can be classified into two types: block ciphers and stream ciphers. A block cipher divides the message or ciphertext into blocks of a fixed size and encrypts or decrypts it one block at a time. A stream cipher encrypts or decrypts one bit at a time. A stream cipher can be seen as a block cipher where the block size is one [17]. Stream ciphers usually require less computing resources than block ciphers; therefore, they are more convenient to provide security on resource-constrained devices. The proposed algorithm in this article is a stream cipher.

On a stream cipher, the encryption transformation consists of: $c_{i}=E_{k_{i}}\left(m_{i}\right)=m_{i}+k_{i}$, and the decryption of: $m_{i}=D_{k_{i}}\left(c_{i}\right)=c_{i}+k_{i}$; where $m_{i}$ is the *i* bit of the message, $c_{i}$ is the *i* bit of the ciphertext and $k_{i}$ is the *i* bit of the keystream. The symbol + represents an addition module two, which is equivalent to the Boolean operation xor, and the keystream consists of a random or pseudo-random number generated by the algorithm from the key. Even though the xor is a simple operation, its use in encryption has been proven secure [18].

As seen in Table 1, the output of the xor is balanced, that is there is a 50% probability of obtaining as output a zero or a one. When encrypting, no assumption can be made about the content of a message, so the value of $c_{i}$ depends on the value of $k_{i}$. If $k_{i}$ is random and unpredictable (50% probability of being zero or one), then $c_{i}$ is also random and unpredictable.

Accordingly, the security of a stream cipher relies on the randomness and unpredictability of the keystream [18]. Randomness can be characterized as a probabilistic property, and statistical tests can be used to evaluate if a sequence has the properties that a truly random sequence has. The statistical tests evaluate the uniformity of the output and determine the presence of patterns that would reveal its non-randomness. Each test examines the sequence for a different type of pattern or property, so a single test cannot be considered enough [19]. To be certain that the results obtained with the statistical tests when evaluating a sequence will be reproducible for any other sequence produced with the same generator, the evaluating sequence has to be very long, such that any pattern or non-random property that the generator produces is revealed in it.

For cryptography, random or pseudo-random sequences [20,21] have to be unpredictable to be considered secure. This means that given an output $k_{i},k_{i+1},\ldots ,k_{n-1}$, there does not exist a polynomial time algorithm that can predict the next bit $k_{n}$ nor the preceding bit $k_{i-1}$ with a probability greater than 50% [18].

### 2.2. Salsa20 Cipher

The GBPA algorithm is based on the Salsa20 cipher designed by Daniel J. Bernstein. Salsa20 consists of a hash function executed ten times in counter mode over an input of 64 bytes and returns an output of the same size. The hash function receives as input a key of 16 or 32 bytes, a nonce of 8 bytes, a counter of 8 bytes and 16 bytes of constant values. Salsa20 is comprised of four functions: quarterround, rowround, columnround and doubleround. The core function is quarterround; doubleround includes all the functions and is executed ten times [22,23,24].

Given $y=(y_{0},y_{1},y_{2},y_{3})$, where each element of *y* is 32 bit long, then $quarterround\left(y\right)=\left(z_{0},z_{1},z_{2},z_{3}\right)$, where:

(1) $$\begin{array}{c} z_{1}=y_{1}⊕\left(\left(y_{0}+y_{3}\right)<<<7\right)\\ z_{2}=y_{2}⊕\left(\left(z_{1}+y_{0}\right)<<<9\right)\\ z_{3}=y_{3}⊕\left(\left(z_{2}+z_{1}\right)<<<13\right)\\ z_{0}=y_{0}⊕\left(\left(z_{3}+z_{2}\right)<<<18\right) \end{array}$$

where symbol a<<<b represents the rotation of value *a* by *b* positions to the left, a+b is arithmetic addition of *a* and *b* and a⊕b represents a bitwise xor between values *a* and *b*.

Function rowround is defined as $rowround\left(y\right)=\left(z_{0},z_{1},z_{2},z_{3},\ldots ,z_{15}\right)$. Given $y=(y_{0},y_{1},y_{2},y_{3},\ldots ,y_{15})$ where *y* is a sequence of 16 elements of 32 bits each, then rowround(y) is also a sequence of 16 elements of 32 bits each.

(2) $$\begin{array}{c} \left(z_{0},z_{1},z_{2},z_{3}\right)=quarterround\left(y_{0},y_{1},y_{2},y_{3}\right)\\ \left(z_{5},z_{6},z_{7},z_{4}\right)=quarterround\left(y_{5},y_{6},y_{7},y_{4}\right)\\ \left(z_{10},z_{11},z_{8},z_{9}\right)=quarterround\left(y_{10},y_{11},y_{8},y_{9}\right)\\ \left(z_{15},z_{12},z_{13},z_{14}\right)=quarterround\left(y_{15},y_{12},y_{13},y_{14}\right) \end{array}$$

Arranging the input $\left(y_{0},y_{1},y_{2},y_{3},\ldots ,y_{15}\right)$ as the square matrix in (3), the function rowround modifies one row on each invocation of quarterround.

(3) $$\left[\begin{array}{cccc} y_{0} & y_{1} & y_{2} & y_{3}\\ y_{4} & y_{5} & y_{6} & y_{7}\\ y_{8} & y_{9} & y_{10} & y_{11}\\ y_{12} & y_{13} & y_{14} & y_{15} \end{array}\right]$$

Given $x=(x_{0},x_{1},x_{2},x_{3},\ldots ,x_{15})$, $columnround\left(x\right)=\left(y_{0},y_{1},y_{2},y_{3},\ldots ,y_{15}\right)$, where *x* is a sequence of 16 elements of 32 bits each, then columnround(x) is also a sequence of 16 elements of 32 bits each.

(4) $$\begin{array}{c} \left(y_{0},y_{4},y_{8},y_{12}\right)=quarterround\left(x_{0},x_{4},x_{8},x_{12}\right)\\ \left(y_{5},y_{9},y_{13},y_{1}\right)=quarterround\left(x_{5},x_{9},x_{13},x_{1}\right)\\ \left(y_{10},y_{14},y_{2},y_{6}\right)=quarterround\left(x_{10},x_{14},x_{2},x_{6}\right)\\ \left(y_{15},y_{3},y_{7},y_{11}\right)=quarterround\left(x_{15},x_{3},x_{7},x_{11}\right) \end{array}$$

Arranging the input $\left(x_{0},x_{1},x_{2},x_{3},\ldots ,x_{15}\right)$ as the square matrix in (5), the function columnround modifies one column on each invocation of quarterround.

(5) $$\left[\begin{array}{cccc} x_{0} & x_{1} & x_{2} & x_{3}\\ x_{4} & x_{5} & x_{6} & x_{7}\\ x_{8} & x_{9} & x_{10} & x_{11}\\ x_{12} & x_{13} & x_{14} & x_{15} \end{array}\right]$$

Function doubleround consists of executing over the input columnround and then rowround:

(6) doubleround(x)=rowround(columnround(x))

The Salsa20 algorithm is defined as $Salsa20\left(x\right)=x+doubleround^{10}\left(x\right)$.

## 3. GBPA Cipher

The proposed algorithm consists of the function GBPA. It receives an input of 19 bytes corresponding to the key, nonce and counter and returns an output of 16 bytes. It comprises two phases: initialization and generation. On initialization, the input parameters plus some constant values are combined and reduced to 16 bytes long and arranged in an appropriate order for the next phase. The generation phase consists first of saving a copy of the init state returned by the previous phase, then executing the quarterround function ten times over the input and finally adding to it the saved init state. The output of this phase is the keystream used to perform the xor with the message.

The function GBPA is defined as $GBPA(k,v,c,T_{0},T_{1})$, where:

$k={\left\{0,1\right\}}^{96}$ is the key,

$v={\left\{0,1\right\}}^{32}$ is the nonce,

$c={\left\{0,1\right\}}^{24}$ is the counter and

$T_{0}$ and $T_{1}$ are the constant values: $T_{0}=\left(84,48\right)$ and $T_{1}=\left(116,49\right)$, corresponding to ASCII values *T0* and *t1*, respectively.

Each execution of the GBPA functions produces a pseudo-random sequence of 16 bytes. A longer sequence can be generated by executing the function in counter mode, which consist of attaching to the nonce a counter that is incremented for each 16-byte block generated, as seen below:

(7) $$GBPA_{k}\left(v,0\right)||GBPA_{k}\left(v,1\right)||\ldots ||GBPA_{k}\left(v,i\right)||\ldots ||GBPA_{k}\left(v,2^{24}-1\right)$$

As shown above, the counter is three bytes long; therefore, the GBPA can generate a sequence of a maximum of $128x2^{24}$ bits per nonce.

Given that $T_{0}=\left(T0_{1},T0_{0}\right)$, $T_{1}=\left(T1_{1},T1_{0}\right)$, $k=k_{11},k_{10},\ldots ,k_{0}$, $v=v_{3},v_{2},v_{1},v_{0}$, $c=c_{2},c_{1},c_{0}$ and $r=r_{2},r_{1},r_{0}$, where the symbology a>>>b represents the rotation of value *a* by *b* positions to the right and a<<<b the rotation of value *a* by *b* positions to the left. The function GBPA is defined as:

(8) $$\begin{array}{c} r_{3}=v_{3}+c_{0}+T1_{1}\\ r_{2}=v_{2}+c_{1}+T1_{0}\\ r_{1}=v_{1}+c_{2}+T0_{1}\\ r_{0}=v_{0}+k_{11}+T0_{0}\\ r=r+(r>>>8) \end{array}$$

Given that $y=(y_{0},y_{1},y_{2},y_{3})$, where $y_{n}={\left\{0,1\right\}}^{32}$; and $z=(z_{0},z_{1},z_{2},z_{3})$, where $z_{n}={\left\{0,1\right\}}^{32}$:

(9) $$\begin{array}{c} y_{0}=\left(k_{2},k_{1},k_{0},r_{0}\right)\\ y_{1}=\left(r_{2},k_{5},k_{4},k_{3}\right)\\ y_{2}=\left(k_{8},k_{7},k_{6},r_{1}\right)\\ y_{3}=\left(r_{3},k_{11},k_{10},k_{9}\right) \end{array}$$

The initial state of *y* is saved and denominated *x*. The quarterround function of Salsa20 is then executed 10 times. In the first nine rounds, *y* is updated by y=z. Finally, to make the function non-invertible, the initial state *x* is added to *z*:

(10) z=x+(z<<<8)

The output of GBPA is *z* of Equation (10), which is the keystream generated from the key.

## 4. Results

### 4.1. Statistical Test

Three statistical test suites were used to evaluate the output of the GBPA cipher: the Statistical Test Suite from NIST (STS) [19], DIEHARD from G. Marsaglia [25] and EACirc from the Centre for Research on Cryptography and Security [26]. The first one contains 15 tests and the second one 19, where each test inspects either the distribution of ones and zeroes, harmonics or patterns in the sequence. The EACirc suite, different from the others, does not have a fixed number of tests, but it builds the tests empirically based on the sequence to be evaluated and a truly random sequence. EACirc is an open-source project available at [27]. The STS and DIEHARD test batteries are well known and have been used by many articles to assess randomness; however, there exists a documented case where these batteries were not able to detect non-randomness on a sequence, while EACirc did [26]. Considering this limitation of STS and DIEHARD, the EACirc framework was also used to evaluate the randomness of GBPA. When performing the evaluation, the three test suites returned a *p*-value that represents the evidence against the null hypothesis that the sequence is random.

As mentioned before, Salsa20 is considered a secure stream cipher by eSTREAM; therefore, the evaluations of the randomness and unpredictability of the GBPA output was done to ensure that the modifications did not make it insecure. The order of presentation of the results of the tests was STS, DIEHARD and finally EACirc.

An implementation in C language of the algorithm was made to obtain the bitstream for the statistical evaluation. Each execution of the algorithm produced a 128-bit output, and after eight million executions, a sequence of $1.024\times 10^{9}$ bits long was obtained, which conforms with the required input size of the test batteries. This output was obtained from feeding the algorithm with “weak parameters”, that is parameters with a very close relationship between them. The purpose of this was to be sure that the algorithm was capable of producing pseudo-random sequences even when its inputs were not random. The pseudo code to generate the bitstream is given in Algorithm 1.

**Algorithm 1:** Generate binary sequence.

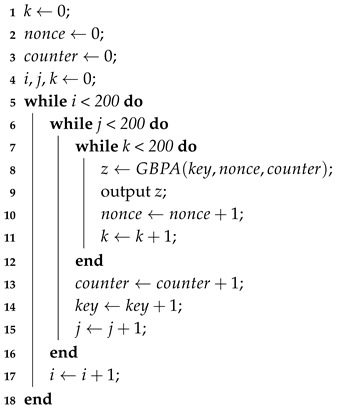



#### 4.1.1. STS

According to [19], the evaluation has to be done for at least 1000 binary sequences, and each statistical test requires a minimum bit size per sequence, where one million bits per sequence is appropriated for all of the tests. Thus, the $1.024\times 10^{9}$ generated bits were fed to STS as 1024 sequences of one million bits each.

The default parameters were used on each test, including the significance level α of 0.01. If the returned *p*-value ≥α, then there was no evidence to reject the null hypothesis, that is the sequence was accepted as random; if the *p*-value < α, then the null hypothesis was rejected, considering the sequence as non-random.

Table 2 shows a typical output after evaluating one sequence. Table 3 shows the proportion of the 1024 sequences that passed each statistical test.

A confidence interval was calculated to determine if the proportion of sequences that passed the statistical tests was within the acceptable range, defined as [19]:

(11) $$CI=p\pm z_{c}\surd \left(p\left(1-p\right)/n\right)$$

where:

p=1−α,

$z_{c}=3$,

n=1024.

CI = 0.99±0.009328007

**Figure 1:**
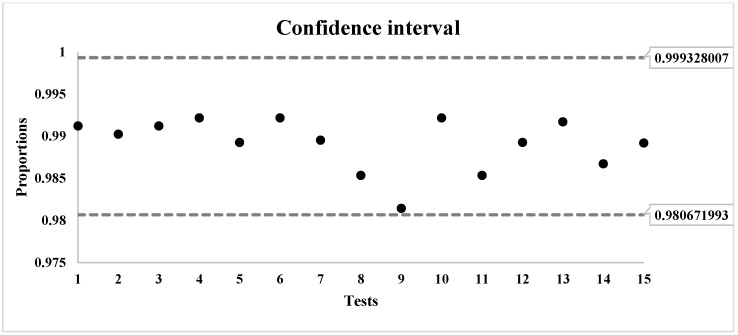
Confidence interval of the STS statistical tests.

Figure 1 shows the proportion of sequences that passed each test; the proportions were within the inferior confidence interval of 0.980671993 and the superior one of 0.999328007.

For reference, the tests were also performed on a bitstream obtained with the algorithm Salsa20, using the same criteria to generate it as with the GBPA cipher. The implementation in C language of Salsa20 is the one in [15]. Table 4 shows the evaluation results.

Of the 1024 sequences of Salsa20, 98.9635% passed the STS statistical tests. Of the 1024 sequences of GBPA, 98.9136% passed the tests. The GBPA cipher has a diminution of 0.0499 compared with the results of Salsa20.

#### 4.1.2. DIEHARD

For DIEHARD, the recommended input size of the battery is a sequence at least 80 million bits long; the generated bitstream conforms with this recommendation.

Results of the evaluation are presented in Table 5. When more than one *p*-value was returned by a test, a final *p*-value was obtained through the Kolmogorov–Smirnov test, which determines the uniformity of multiple *p*-values.

A Kolmogorov–Smirnov test was used again to determine the uniformity of all the *p*-values returned by the tests shown in Table 5. The result was a *p*-value of 0.262431.

As was done previously, DIEHARD tests were also performed on the binary sequence generated with Salsa20. Table 6 presents the results. The *p*-value returned by the Kolmogorov–Smirnov test comprised of all the *p*-values in Table 6 was 0.184669.

#### 4.1.3. EACirc

One of the advantages of EACirc is that it requires a lower amount of bits to detect non-randomness compared with the previous test suites, being able to work with even 1000 bits [26]. The parameters used during the evaluation of GBPA and Salsa20 sequences were the default ones presented in [27], which can be summarized as follows:

α: 0.01,

Number of epochs: 300,

Test vector size: 16,

Test vector count: 1000,

Function set: NOP, CONS, NOT, AND, NAND, OR, XOR, NOR, SHIL, SHIR, ROTL, ROTR, MASK.

The function set parameter included all the operations supported by EACirc, and these were used to construct the test stochastically using a genetic algorithm [26].

In Table 7, the results of GBPA and Salsa20 are presented. The shown *p*-values corresponded to the Kolmogorov–Smirnov test performed by EACirc comprised of the *p*-values returned by it. As can be seen in Table 7, the bitstreams generated by GBPA and Salsa20 were considered random by the EACirc suite.

### 4.2. Computing Requirements

The GBPA and Salsa20 ciphers were implemented on the 8-bit AVR microcontroller Atmega644p [28] using the Integrated Development Environment (IDE) Atmel Studio 7 [29] of Microchip, with the compiler and linker AVR/GNU Version 5.4.0. With this IDE, the memory requirements and executing cycles of both algorithms were retrieved.

**Figure 2:**
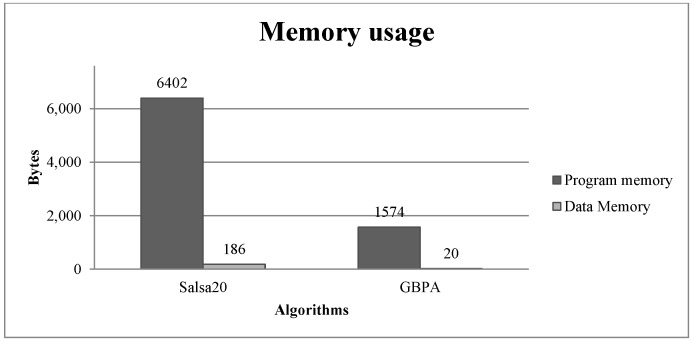
Memory footprint comparison between GBPA and Salsa20.

In Figure 2, a comparison of the usage of data and program memory of the algorithms is presented. The information was returned by the compiler and linker AVR/GNU, and no dynamic memory allocation was done in the implementations.

**Figure 3:**
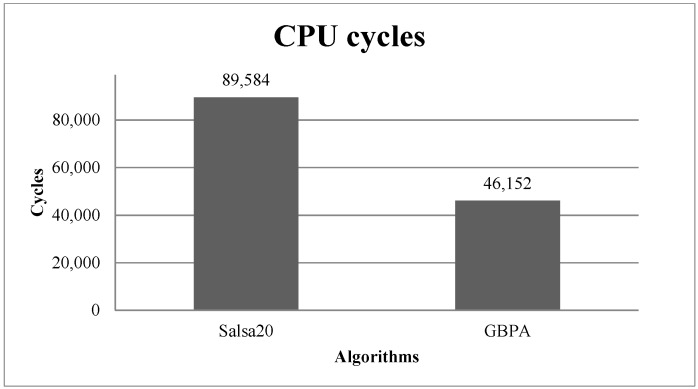
Processor cycle comparison between Salsa20 and GBPA, when generating an output 512 bits long.

The number of processor cycles required to execute the algorithms was obtained using the Microchip AVR MCU Simulator. This tool is considered accurate because it uses models based on the register-transfer level (RTL) code used in the making of the actual microcontroller [30]. As indicated in Section 2 and Section 3, the output size of Salsa20 was 512 bits, and the output of GBPA was 128 bits. Both algorithms had to produce the same output size to compare their number of cycles, so GBPA was executed four times. Figure 3 presents the comparison of cycles necessary to generate an output of 512 bits.

When measuring the required processor cycles of an algorithm, the amount of consumed energy by it can be calculated with (12). Table 8 shows the power consumption of the ciphers in a system operating at 2 V, 0.5 mA, low-power mode running at 1 MHz. As can be seen, GBPA consumed 48.4819% less power than Salsa20.

(12) $$CE=\frac{W}{f_{CPU}}\times PC$$

where:

CE= consumed energy,

W= Watts,

$f_{CPU}=$ frequency of the processor clock,

PC= processor cycles used by the algorithm.

GBPA algorithm required 75.4139% less program memory, 89.2473% less data memory, 48.4819% fewer executing cycles and 48.4819% less power than Salsa20.

A comparison of the computing requirements of GBPA against lightweight block ciphers is presented in Table 9. The requirements were from implementations of the algorithms on 8-bit AVR microcontrollers in C language. The memory footprint and processor cycles of the HIGHT, RC5 and Skipjack ciphers were obtained from [31] and of the PRESENT cipher from [32].

As shown in Table 9, the GBPA cipher had lower program and data memory requirements than all the ciphers and used fewer executing cycles than HIGHT, RC5 and Skipjack. PRESENT used 48.5699% fewer executing cycles than GBPA; however, it used 58.7526% more program memory and 92.1875% more data memory. Fewer executing cycles means a higher-throughput; however, this was being achieved at the cost of a higher requirement of memory, a limited resource on IoT devices. A low memory requirement allows a device to be implemented as small and low-cost, both of them essential characteristics of IoT because it enables ubiquitous computing. GBPA had lower program and data memory requirements and a proper throughput when comparing it with Salsa20 and the rest of the lightweight ciphers, making it appropriate for the restrictions and characteristics of IoT devices.

## 5. Discussion

As mentioned above, to be part of the eSTREAM portfolio, Salsa20 was under evaluation for four years (2004 through 2008), and no possible attack on it was detected [15]; weaknesses were found only when using a reduced number of rounds [33,34,35]; an analysis of the security of the cipher can be seen in [36]. The GBPA cipher uses Salsa20’s core function quarterround, but with fewer parameters to make its use possible on resource-constrained devices. Evaluation of the randomness and unpredictability was performed on its output to evaluate that the modifications were secure. As explained in [18], the xor operation returns a random output when at least one of its inputs is random, and the random part in a stream cipher is the keystream generated by the algorithm. Three statistical test suites were used to evaluate the keystream: the first one was STS, which was designed to evaluate bitstreams for cryptographic applications; the second one was DIEHARD, a well-known test battery; and EACirc was the third one. As mentioned before, there is a documented case were the STS and DIEHARD test batteries were not able to detect non-randomness in a sequence, while EACirc was capable of such; consequently, the EACirc suite of empirical tests was also used.

The mentioned test batteries were used to evaluate a $1.024\times 10^{9}$ bitstream. When dividing the bitstream into 1024 sequences and inputting them into STS, 98.9136% passed the tests; some sequences did not pass some them, but the proportion that passed was within the confidence interval. There are expected to be generated random sequences that do not pass some tests because if they do, this means that its generator is not capable of generating any sequence and its output is not uniform. As shown in Table 10, compared with Salsa20, there was a decrease of 0.0499% of sequences that passed the tests, which does not seem significant. When evaluating the bitstream with DIEHARD, a *p*-value of 0.262431 was obtained, which shows that there is no evidence of the bitstream not being random, that is the test battery could not distinguish it from a truly random sequence. the EACirc suite was also used for the evaluation of the output of GBPA, and a *p*-value of 0.515966 was returned by it, showing the acceptance of the null hypothesis. Both Salsa20 and GBPA sequences were accepted as random by DIEHARD and EACirc, but GBPA obtained a higher *p*-value, which means lower evidence against the null hypothesis that the sequence is random; the difference in *p*-values is presented in Table 10. The results of STS, DIEHARD and EACirc show that the keystream generated by GBPA is indistinguishable from random, so after performing the xor with a message to cipher, the output will also be indistinguishable from random.

Regarding its computing requirements, GBPA uses little more than 1.5 KB of program memory and only 20 bytes of data memory; therefore, it would not have an impact on memory usage on the system. Table 11 shows how many more resources the algorithms Salsa20, HIGHT, PRESENT, RC5 and Skipjack use compared to GBPA. As can be seen, GBPA uses less memory and processor cycles than the compared algorithms, except for the PRESENT cipher, which uses fewer processor cycles. As can also be seen, this is achieved at the cost of more memory usage. Considering that IoT devices have limited memory and that it has to be divided between the application and network services, besides the security services, the higher memory requirement by the cipher can have a high impact on the device, or its use might not be feasible. GBPA has lower memory usage and proper processor time usage when comparing it with Salsa20 and lightweight ciphers.

The proposed algorithm is not intended to replace Salsa20 or traditional ciphers; it is recommended to use them when the device’s resources allow it. Instead, a security solution is being provided to the many devices that could not afford those algorithms, such as small smart sensors and other low-cost IoT devices.

## 6. Conclusions

IoT is a promising technology that could bring a significant improvement to our daily lives, from making our lives more comfortable to having a better response to emergency situations. Even though it has many advantages, its characteristics make it not only very vulnerable to attacks, but also, such attacks can have severe consequences in the system and even for the user. Encryption is a security primitive that could prevent some of the attacks, but because of the limited resources in IoT devices of Class 0, the use of traditional security algorithms is not viable. In this document, the stream cipher GBPA designed for IoT has been presented. The algorithm is based on the Salsa20 cipher, which was under evaluation by the international community, who decided that it was secure enough to be part of the eSTREAM project. GBPA uses Salsa20’s core function, but with fewer input parameters, a smaller memory footprint and lower processor-time usage. The GBPA cipher has a small output size; this is appropriate for IoT devices because the information sent by them is usually contextual data in the form of short-length packets; thus, no unnecessary memory and processor usage is performed. When comparing the computing requirements of GBPA against lightweight ciphers, GBPA resulted in lower program and data memory usage. The low program and data memory and processor-time usage allow low-cost resource-constrained IoT devices of Class 0 to be able to implement a security service that they are not currently capable of, to protect the user’s data privacy, the user’s welfare and the system from attacks that could damage or disrupt its functionality.

The randomness of the output of the cipher was evaluated using three statistical test suites: STS, which was designed to assess pseudo-random numbers for cryptographic applications, the well-known DIEHARD test battery and EACirc, an empirical test suite, which in some cases can provide better results than the previous two. With the three of them, good results were obtained. The tests were also applied to Salsa20 for reference, and no significant difference between the results of the two algorithms was found.

As future work, a modification of the algorithm is planned to make it support two key sizes, the current 96-bit key and also a 128-bit key; the latter for devices with more computing resources and security requirements.

## Figures and Tables

**Table 1:** xor truth table.

$m_{i}$	$k_{i}$	$c_{i}$
0	0	0
0	1	1
1	0	1
1	1	0

**Table 2:** Typical results when evaluating one sequence generated by Generador de Bits Pseudo Aleatorios (GBPA) using STS.

Test	*p*-Value	Result
1. Frequency	0.480188	SUCCESS
2. Frequency within a Block	0.979526	SUCCESS
3. The Runs	0.133743	SUCCESS
4. Longest-Run-of-Ones in a Block	0.816729	SUCCESS
5. Binary Matrix Rank	0.845216	SUCCESS
6. Discrete Fourier Transform	0.378341	SUCCESS
7. Non-overlapping Template Matching	0.516262	SUCCESS
8. Overlapping Template Matching	0.697754	SUCCESS
9. Maurer’s “Universal Statistical”	0.953444	SUCCESS
10. Linear Complexity	0.671206	SUCCESS
11. Serial	0.697067	SUCCESS
12. Approximate Entropy	0.677134	SUCCESS
13. Cusums	0.604715	SUCCESS
14. Random Excursions	0.57388	SUCCESS
15. Random Excursions Variant	0.382226	SUCCESS

**Table 3:** Proportion of the 1024 sequences generated by GBPA that passed each STS test.

Test	Proportion
1. Frequency	0.991211
2. Frequency within a Block	0.990234
3. The Runs	0.991211
4. Longest-Run-of-Ones in a Block	0.992188
5. Binary Matrix Rank	0.989258
6. Discrete Fourier Transform	0.992188
7. Non-overlapping Template Matching	0.989522
8 Overlapping Template Matching	0.985352
9. Maurer’s “Universal Statistical”	0.981445
10. Linear Complexity	0.992188
11. Serial	0.985352
12. Approximate Entropy	0.989258
13. Cusums	0.9917
14. Random Excursions	0.986736
15. Random Excursions Variant	0.989193

**Table 4:** Proportion of the 1024 sequences generated by Salsa20 that passed each STS test.

Test	Proportion
1. Frequency	0.991211
2. Frequency within a Block	0.995117
3. The Runs	0.988281
4. Longest-Run-of-Ones in a Block	0.981445
5. Binary Matrix Rank	0.988281
6. Discrete Fourier Transform	0.988281
7. Non-overlapping Template Matching	0.990228
8. Overlapping Template Matching	0.992188
9. Maurer’s “Universal Statistical”	0.992188
10. Linear Complexity	0.992188
11. Serial	0.990234
12. Approximate Entropy	0.989258
13. Cusums	0.990234
14. Random Excursions	0.986388
15. Random Excursions Variant	0.989008

**Table 5:** *p*-values returned by DIEHARD tests when evaluating a sequence of $1.024\times 10^{9}$ bits generated by GBPA.

Test	*p*-Value	Result
1. Birthday spacings	0.432844	SUCCESS
2. Overlapping 5-permutation	0.402562	SUCCESS
3. Binary rank for 31 × 31 matrices	0.622226	SUCCESS
4. Binary rank for 32 × 32 matrices	0.573546	SUCCESS
5. Binary rank for 6 × 8 matrices	0.437927	SUCCESS
6. Bitstream	0.584245	SUCCESS
7. OPSO	0.964164	SUCCESS
8. OQSO	0.645856	SUCCESS
9. DNA	0.332053	SUCCESS
10. Count-the-1’s on a stream of bytes	0.246727	SUCCESS
11. Count-the-1’s for specific bytes	0.754247	SUCCESS
12. Parking lot	0.17368	SUCCESS
13. Minimum distance	0.656646	SUCCESS
14. 3D spheres	0.803322	SUCCESS
15. Squeeze	0.363291	SUCCESS
16. Overlapping sums	0.327737	SUCCESS
17. Runs up	0.448923	SUCCESS
18. Runs down	0.636782	SUCCESS
19. Craps	0.881032	SUCCESS

**Table 6:** *p*-values returned by DIEHARD tests when evaluating a sequence of $1.024\times 10^{9}$ bits generated by Salsa20.

Test	*p*-Value	Result
1. Birthday spacings	0.194329	SUCCESS
2. Overlapping 5-permutation	0.526502	SUCCESS
3. Binary rank for 31 × 31 matrices	0.320943	SUCCESS
4. Binary rank for 32 × 32 matrices	0.348652	SUCCESS
5. Binary rank for 6 × 8 matrices	0.646051	SUCCESS
6. Bitstream	0.855505	SUCCESS
7. OPSO	0.647555	SUCCESS
8. OQSO	0.193828	SUCCESS
9. DNA	0.450855	SUCCESS
10. Count-the-1’s on a stream of bytes	0.480096	SUCCESS
11. Count-the-1’s for specific bytes	0.669009	SUCCESS
12. Parking lot	0.386253	SUCCESS
13. Minimum distance	0.705886	SUCCESS
14. 3D spheres	0.670799	SUCCESS
15. Squeeze	0.420715	SUCCESS
16. Overlapping sums	0.505699	SUCCESS
17. Runs up	0.606394	SUCCESS
18. Runs down	0.393157	SUCCESS
19. Craps	0.362921	SUCCESS

**Table 7:** *p*-values returned by the EACirc framework when evaluating sequences generated by Salsa20 and GBPA.

Algorithm	*p*-Value	Result
Salsa20	0.375193	SUCCESS
GBPA	0.515966	SUCCESS

**Table 8:** Power consumption comparison between Salsa20 and GBPA.

Algorithm	Power Consumption (mJ)
Salsa20	0.089584
GBPA	0.046152

**Table 9:** Computing requirement comparison between lightweight block ciphers and GBPA.

Algorithm	Program Memory (Bytes)	Data Memory (Bytes)	CPU Cycles for a 512-bit Output
HIGHT	3906	584	514,840
PRESENT	3816	256	23,736
RC5	3188	72	565,600
Skipjack	5020	328	139,120
GBPA	1574	20	46,152

**Table 10:** Comparison of the results of STS and DIEHARD between Salsa20 and GBPA.

Tests	Salsa20	GBPA	Difference
STS (percentage of approved sequences)	98.9635%	98.9136%	−0.0499
DIEHARD (*p*-value)	0.184669	0.262431	0.077762
EACirc (*p*-value)	0.375193	0.515966	0.140773

**Table 11:** Difference in the percentage of computing resource utilization of the algorithms against GBPA.

Parameter	Salsa20	HIGHT	PRESENT	RC5	Skipjack
Program memory	−75.4139%	−59.7030%	−58.7526%	−50.6274%	−68.6454%
Data memory	−89.2473%	−96.5753%	−92.1875%	−72.2220%	−93.9024%
CPU cycles	−48.4819%	−91.0357%	+48.5699%	−91.8402%	−66.8258%
